# Bihemispheric Motor Cortex Transcranial Direct Current Stimulation Improves Force Steadiness in Post-Stroke Hemiparetic Patients: A Randomized Crossover Controlled Trial

**DOI:** 10.3389/fnhum.2016.00426

**Published:** 2016-08-23

**Authors:** Rafael A. Montenegro, Adrian Midgley, Renato Massaferri, Wendell Bernardes, Alexandre H. Okano, Paulo Farinatti

**Affiliations:** ^1^Graduate Program in Clinical and Experimental Physiopathology, Faculty of Medical Sciences, University of Rio de Janeiro StateRio de Janeiro, Brazil; ^2^Laboratory of Physical Activity and Health Promotion, Institute of Physical Education and Sports, University of Rio de Janeiro StateRio de Janeiro, Brazil; ^3^Department of Sport and Physical Activity, Edge Hill UniversityOrmskirk, Lancashire, UK; ^4^Physical Education Department, Federal University of Rio Grande do NorteNatal, RN, Brazil

**Keywords:** motor cortex, performance, physical rehabilitation, strength, stroke, tDCS

## Abstract

Post-stroke patients usually exhibit reduced peak muscular torque (PT) and/or force steadiness during submaximal exercise. Brain stimulation techniques have been proposed to improve neural plasticity and help to restore motor performance in post-stroke patients. The present study compared the effects of bihemispheric motor cortex transcranial direct current stimulation (tDCS) on PT and force steadiness during maximal and submaximal resistance exercise performed by post-stroke patients vs. healthy controls. A double-blind randomized crossover controlled trial (identification number: TCTR20151112001; URL: http://www.clinicaltrials.in.th/) was conducted involving nine healthy and 10 post-stroke hemiparetic individuals who received either tDCS (2 mA) or sham stimulus upon the motor cortex for 20 min. PT and force steadiness (reflected by the coefficient of variation (CV) of muscular torque) were assessed during unilateral knee extension and flexion at maximal and submaximal workloads (1 set of 3 repetitions at 100% PT and 2 sets of 10 repetitions at 50% PT, respectively). No significant change in PT was observed in post-stroke and healthy subjects. Force steadiness during knee extension (~25–35%, *P* < 0.001) and flexion (~22–33%, *P* < 0.001) improved after tDCS compared to the sham condition in post-stroke patients, but improved only during knee extension (~13–27%, *P* < 0.001) in healthy controls. These results suggest that tDCS may improve force steadiness, but not PT in post-stroke hemiparetic patients, which might be relevant in the context of motor rehabilitation programs.

## Introduction

Post-stroke patients often exhibit motor sequels (Langhorne et al., 2011) and hemiparesis (Prado-Medeiros et al., 2012) that are associated with increased variability in the application of force during motor tasks (Chow and Stokic, 2011). This condition typically results in low force steadiness (Moritz et al., 2005) and poor movement control (Kornatz et al., 2005) that can negatively impact on the ability to perform activities of daily living (Timmermans et al., 2014). Patients affected by stroke show a relative imbalance in either transcallosal inhibition or inter-hemispheric cerebral excitability, with hypo-excitability of the affected motor cortex concomitant to hyper-excitability of the non-affected motor cortex (Murase et al., 2004; Bolognini et al., 2011). Strategies to help counteract these imbalances and improve neural plasticity should therefore be beneficial for those patients (Bolognini et al., 2011; Simonetta-Moreau, 2014). Previous studies reported that improvements in neuronal plasticity and functional ability could be optimized by combining physical exercise and neurological therapy (Langhorne et al., 2011; Mang et al., 2013; Billinger et al., 2014).

Non-invasive brain stimulation techniques, such as transcranial magnetic stimulation (TMS) and transcranial direct current stimulation (tDCS) have been considered as promising tools for restoring motor control and performance in post-stroke patients (Bolognini et al., 2011). Recently, Tanaka et al. (2011) demonstrated that a unilateral anodal tDCS over motor leg cortex slightly enhanced the maximal force production of the paretic leg. Even though there is controversial findings (O’Shea et al., 2014), evidence indicates that bihemispheric motor cortex tDCS seems to be more effective than unilateral tDCS (i.e., anodal or cathodal tDCS) to increase motor-evoked potentials in upper and lower limb contralateral to the affected cortex, thereby improving neuroplasticity (Bolognini et al., 2009; Cha et al., 2014) and to decrease excitability in regions that inhibit those areas (Vines et al., 2008). In addition, studies with post-stroke patients have evaluated the effects of tDCS using relatively restricted motor tasks, as isometric grip strength and hand function (Khedr et al., 2013; Cha et al., 2014). Thus, the effects of tDCS on the performance of tasks demanding submaximal and maximal strength, and force steadiness during exercise involving larger muscle groups of the legs are yet to be determined. This would be useful, since the muscle strength of both lower limbs is related to activities of daily living.

Recent studies with healthy subjects failed to observe changes in motor performance in response to tDCS in both upper (Hendy and Kidgell, 2013) and lower limbs extremities (Montenegro et al., 2015), which may be due to a possible “ceiling effect” when motor neuronal excitability is already optimal. This may help to explain the mixed findings in regards to the effects of tDCS upon cortical excitability in healthy subjects vs. post-stroke patients (Suzuki et al., 2012). In brief, it is feasible to think that the effects of tDCS upon cortical excitability rely on the extent to which the cortical function is preserved (Byblow et al., 2015), but there is a lack of research investigating this possibility. Comparisons between post-stroke patients and healthy controls regarding the effects of tDCS upon strength performance and force steadiness during gross motor tasks would be useful to test this hypothesis. Thus, the purpose of the present study was to investigate whether tDCS applied to the affected motor cortex in post-stroke hemiparetic patients would increase the peak muscular torque (PT) and force steadiness during a gross motor task in comparison with healthy controls. We hypothesized that tDCS would be capable to increase PT and force steadiness in post-stroke patients, but not in healthy subjects with preserved cortical function.

## Materials and Methods

### Experimental Approach to the Problem

This randomized crossover study was designed to investigate the effects of tDCS applied in post-stroke hemiparetic patients, upon PT and force steadiness during a gross motor task in comparison with healthy controls. The experimental design of this study included three non-consecutive visits to the laboratory. On the first visit, participants underwent a familiarization protocol with the equipment and procedures to reduce the potential confounding influence of motor learning.

**Figure 1:**
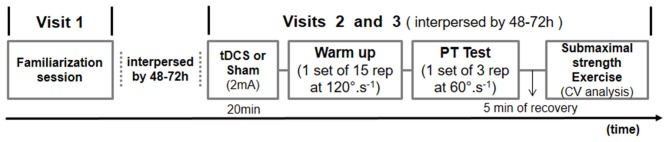
**Schematic overview of the experimental protocol.** tDCS, transcranial direct current stimulation; PT, peak torque; CV, coefficient of variation.

On the second and third visits, 20 min of either tDCS with 2 mA or sham condition were applied in randomized counter-balanced order, as defined by the Latin square. Immediately after receiving tDCS, maximal and submaximal isokinetic unilateral knee extension and flexion exercises were performed. A wash-out period of 48–72 h between visits was applied to avoid tDCS carryover effects. Participants were seated in a moderately lit room with temperature and humidity ranging between 21–23°C and 55–70%, respectively. All measurements were taken between 9–11 a.m. to avoid bias due to circadian variation. Participants were instructed to refrain from consuming ergogenic beverages like coffee and soft drinks within 12 h before the experimental sessions. The Figure 1 summarizes the experimental protocol.

### Subjects

The sample size was predetermined using the GPower version 3.1.2 (Universität Kiel, Kiel, Germany) for an effect size (Cohen’s *d*) of 1.02, α fixed at 0.05, and β error probability set at 0.20. The adopted effect size was based on previous data investigating acute strength increase due to tDCS in healthy individuals and post-stroke patients (Tanaka et al., 2009, 2011). Additional eligibility criteria were: (a) diagnosis of right or left hemiparesis caused by stroke occurring at least 6 months prior to the experiment; (b) Score in Berg’s balance scale compatible with low risk of falling (scores higher than 36 out of 56 points) and 20 on the Fulg-Meyer scale (possible range 0–34 points); and (c) prior participation in the neuromotor rehabilitation program for at least 6 months. Exclusion criteria included: (a) cognitive impairment precluding fully understanding of the protocol instructions or exercise performance; (b) smoking or use of ergogenic substances; (c) presence of implanted devices such as cochlear implants, Internal Pulse Generator or medication pumps; and (d) any additional cardiovascular, respiratory, muscle, neurological, or skeletal problems that prevented the application of tDCS (Rossi et al., 2009; Fregni et al., 2015) or physical exercise. The study gained approval from the institutional ethics committee (process number: 38263114.7.0000.5259) and was registered as clinical trial (identification number: TCTR20151112001). Prior to enrolling in the study, all participants signed written informed consent in accordance with the Declaration of Helsinki.

Volunteers were recruited by advertising in the outpatient clinic of the University Hospital. From the 24 post-stroke patients initially screened, 14 were excluded due to clinical disorders, such as motor and Wernicke aphasia (*n* = 2), uncontrolled cardiac arrhythmia (*n* = 2), carotid stenosis (*n* = 1), uncontrolled hypertension (*n* = 3), advanced gonarthrosis (*n* = 1), biomechanical impairments precluding muscular effort (*n* = 2), and voluntary waiver (*n* = 3). Six volunteers for the control group were excluded due to smoking (*n* = 4) and hypertension (*n* = 2). Therefore, 10 patients (eight males) with chronic subcortical stroke aged 52 ± 14 year, and nine healthy male controls aged 26 ± 7 year were retained to participate in the study. Table 1 summarizes participants’ anthropometric and functional characteristics. According to Berg’s balance and Fulg-Meyer scale scores, the post-stroke group was classified with mild-to-moderate motor impairment.

**Table 1:** **Mean ± SD participant characteristics and functional scores of patients with stroke and healthy controls**.

	**Group**			
	Stroke (*n* = 10)		Healthy (*n* = 9)	
***Participant characteristics***
Age (years)	52	± 14	26	± 7
Height (cm)	170	± 10	173	± 6
Body mass (kg)	77	± 11	73	± 10
**Body mass index (kg/m^2^)**	27	± 3	24	± 3
Time after stroke (month)	24	± 10	–
***Functional Scores***
Berg’s balance scale	47	± 4	NE
**Fulg-Meyer’s functional scale**	28	± 5	NE

*NE, Not evaluated*.

### Procedures

#### Submaximal and Maximal Strength Exercise

Isokinetic strength during submaximal and maximal unilateral concentric knee extension and flexion was tested using a Biodex^TM^ System 4 PRO dynamometer (Biodex Medical Systems Inc., Shirley, NY, USA). The lateral femoral condyle was aligned with the dynamometer’s rotation axis and the cuff of the force transducer was placed approximately 5 cm proximal to the lateral malleoli. The range of motion varied between 0° to 90° with the execution speed fixed at 60°·s^−1^ for submaximal and maximal exercises (Eng et al., 2002). One warm-up set of 15 repetitions with execution speed fixed at 120°·s^−1^ was performed before exercise testing. Subsequently, one set of three maximal repetitions with 120 s intervals between the legs was performed to assess PT. The PT assessment was considered valid only when the coefficient of variation (CV) between repetitions was less than 15% (Eng et al., 2002). The highest PT value within a given set was recorded as the final result. Verbal encouragement was provided during all PT tests.

**Figure 2:**
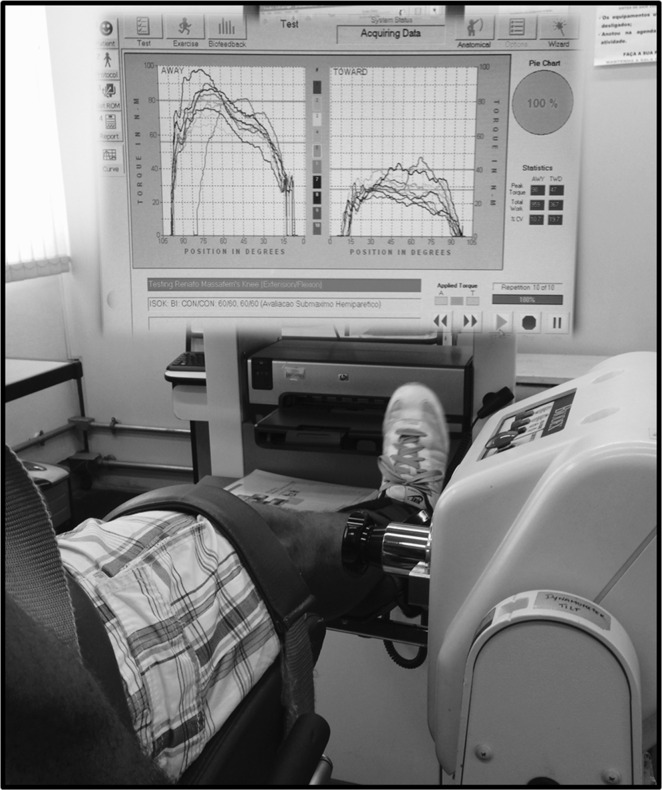
**Illustration of submaximal knee extension and flexion exercise using muscular torque biofeedback**.

After 5 min of recovery, subjects also performed two sets of 10 submaximal repetitions with loads corresponding to 25% and 50% of PT obtained in the maximal exercise. Maximal and submaximal tests were interspersed with rest periods of 10–20 min. The chair settings, range of motion, and speed of movement used in the maximal test protocol were maintained during submaximal exercise. The CV of muscular torque during the exercise was used as a marker of force steadiness. A screen showing reference lines corresponding to 50% (top line) and 25% (bottom line) of PT was fixed in front of the participants, to provide visual feedback during the exercise. Subjects were encouraged to keep the muscular torque within the range defined by the two reference lines (illustrated in Figure 2). In order to compare PT and force steadiness between limbs and groups, the paretic leg in the stroke group and dominant leg in the healthy group were considered as “comparison” legs, and the non-paretic leg in the stroke group and non-dominant leg in the healthy group was considered as “control” legs.

#### Transcranial Direct Current Stimulation (tDCS)

The electric current was applied using a pair of sponges with a surface area of 35 cm^2^, and a pair of electrodes. The sponges were previously soaked in saline solution consisting of 140 mMols of NaCl dissolved in Milli-Q water. Anodal and cathodal electrodes were connected to a constant current stimulation device incorporating three 9 V batteries with a maximal output of 10 mA (Eldith DC-Stimulator, NeuroConn^TM^, Ilmenau, Germany). A bi-cephalic montage was chosen for the electrode positioning (Vines et al., 2008). The anode electrode was placed over the affected motor cortex in patients with stroke and over the contralateral motor cortex of the dominant leg in healthy subjects (i.e., C3 or C4 according to the international EEG 10–20 system). The cathodal electrode was placed over the contralateral motor cortex. A similar electrode positioning was adopted during sham and experimental conditions.

This electrode montage (i.e., C3/C4) has been previously applied by studies using TMS to elicit motor evoked potential in both upper and lower limbs, as described elsewhere (Legatt et al., 2016). In order to blind participants to the experimental conditions, 30 s of tDCS were applied at the start of sham stimulation, in order to induce a slight itching sensation (Gandiga et al., 2006).

### Statistical Analysis

Data normality was confirmed by the Shapiro-Wilk test and therefore results are presented as means ± standard deviations (SD). The effects of condition (sham *vs.* tDCS), limb (control *vs.* comparison), and group (healthy *vs.* stroke) upon PT and force steadiness were compared using Mixed ANOVA procedures. Fixed and random effects were retained in models for each outcome variable when a Wald Test or Likelihood ratio test (as appropriate) was statistically significant. *Post hoc* pair wise comparisons were performed using Bonferonni adjusted *P* values in the event of significant *F* ratios. Distributional assumptions regarding model residuals were checked and verified using standard graphical methods. Two-tailed statistical significance was accepted as *P* ≤ 0.05. All statistical analyses were performed using the IBM SPSS Statistics 22 software (SPSS^TM^ Inc., Chicago, IL, USA).

## Results

**Figure 3:**
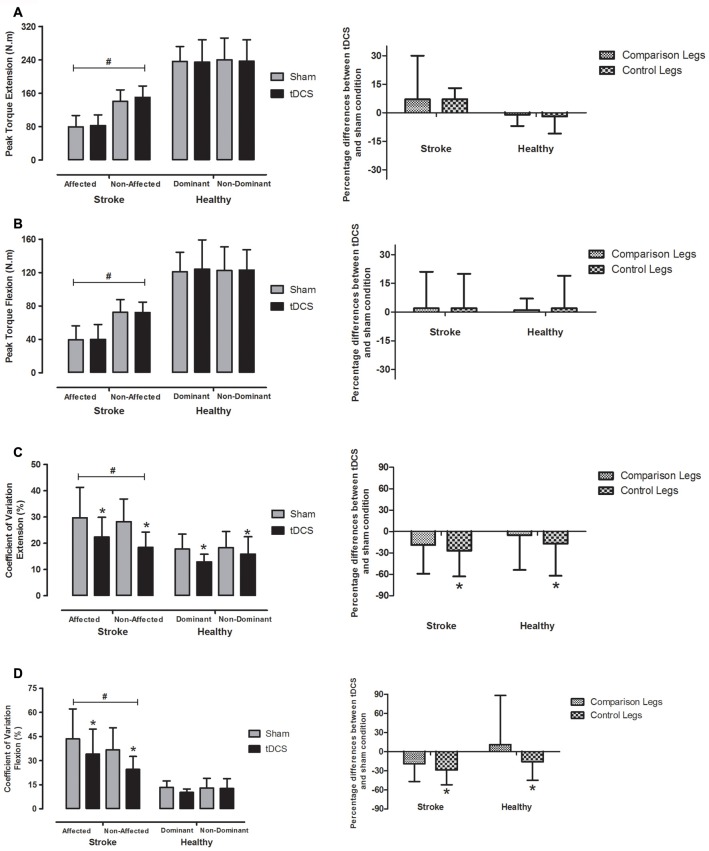
**Mean ± SD peak muscular torque (PT; left side of the lines A and B) during one set of three maximal repetitions and the CV (left side of the lines C and D) representing force steadiness between two sets of 10 repetitions at 50% PT in bihemispheric motor cortex tDCS (black bars) and sham (gray bars) conditions in affected and non-affected legs (stroke group) and dominant and non-dominant legs (healthy group).** Percent differences between tDCS and sham condition in regards to PT and CV are plotted on the right side of the Figure. ^#^Significant difference between stroke and healthy groups (*P* < 0.001). *Bihemispheric tDCS significantly lower than sham condition (*P* < 0.001). tDCS, transcranial direct current stimulation; comparison legs, paretic leg in stroke group and dominant leg in healthy group; control legs, non-paretic leg in stroke group and non-dominant leg in healthy group.

Figures 3A–D (left side) exhibit PT and force steadiness represented by CV after tDCS and sham conditions, in both “control” (non-affected leg in stroke patients and non-dominant leg in healthy subjects) and “comparison” (affected leg in stroke patients and dominant leg in healthy subjects) limbs. On the right side, percent differences between tDCS and sham conditions in regards to PT and CV were plotted. No group *vs.* condition interaction was observed in PT for either knee extension (*F* = 1.3, *P* = 0.26) or flexion (*F* = 0.1, *P* = 0.76). Group *vs.* limb interaction was observed for PT during knee extension (*F* = 63.7, *P* < 0.001) and flexion (*F* = 34.9, *P* < 0.001). As expected, the post-stroke group showed lower PT in “comparison” *vs.* “control” leg during both knee extension (mean difference = 64.4 N.m, 95% CI = 53.7–75.0, *P* < 0.001) and flexion (mean difference = 32.7 N.m, 95% CI = 25.1–40.3, *P* < 0.001). No difference in PT between legs was observed in healthy controls for knee extension (*P* = 0.63) and flexion (*P* = 0.95).

No condition *vs.* group *vs.* limb interaction was found for force steadiness during knee extension (*F* = 0.6, *P* = 0.45) or flexion (*F* = 1.4, *P* = 0.25). However, significant differences between conditions (sham *vs.* tDCS) were observed in regards to force steadiness for knee extension (*F* = 15.4, *P* < 0.001) and flexion (*F* = 10.1, *P* = 0.006), and between groups (hemiparetic *vs.* healthy) for knee extension (*F* = 19.3, *P* < 0.001) and flexion (*F* = 43.5, *P* < 0.001). A significant group *vs.* condition interaction was also detected for knee flexion (*F* = 6.2, *P* = 0.023). As expected, higher CVs and therefore lower force steadiness was observed in post-stroke patients *vs.* healthy controls along knee extension (mean difference = 8.5%, 95% CI = 4.4–12.5, *P* < 0.001) and flexion (mean difference = 20.9%, 95% CI = 14.2–27.5, *P* < 0.001).

No significant interaction was detected between groups and conditions for the force steadiness during knee extension, however, differences between conditions were detected when considering the whole sample (mean difference = 6.3%, 95% CI = 3.1–9.4, *P* < 0.001). The tDCS improved the force steadiness by ~13–35% during the knee extension in both legs and groups. With regard to the knee flexion, interaction between group and condition was found and force steadiness was significantly improved in post-stroke patients (mean difference = 11.4%, 95% CI = 5.6–17.3, *P =* 0.001), but not in healthy controls (mean difference = 1.4%, 95% CI = −4.8 to 7.5, *P =* 0.64).

## Discussion

The main purpose of the present study was to investigate whether motor cortex tDCS would improve PT and force steadiness during a gross motor task, in post-stroke hemiparetic patients compared to healthy controls. The main findings were: (i) Bihemispheric tDCS improved force steadiness in post-stroke hemiparetic patients during unilateral knee extension and flexion, but only during knee extension in healthy controls; and (ii) No change in PT during knee extension or flexion in stroke and healthy groups were detected after tDCS. Therefore, discrepancies in strength between legs and groups were not reduced by tDCS. In brief, tDCS was capable of improving force steadiness during submaximal knee extension and flexion exercise in post-stroke hemiparetic patients, but not the maximal strength reflected by PT during isokinetic exercise.

A decreased PT in paretic compared to non-paretic limb is common in patients after stroke (Eng et al., 2002), which has been acknowledged in those patients as the main factor interfering with voluntary force control in paretic-spastic muscles (Chang et al., 2013). Evidently, this handicap has a negative impact on the ability to perform activities of daily living (Teixeira-Salmela et al., 1999; Timmermans et al., 2014). In the present study, the tDCS did not improve the maximal strength reflected by PT in the paretic limb of post-stroke patients. These data disagree with results from *a prior* study by Tanaka et al. (2011), showing an increase of maximal isometric strength after tDCS during knee extension performed by subcortical post-stroke patients.

These mixed results could be partially explained by differences in experimental designs. Firstly, the tDCS electrode positioning presently applied was bihemispheric (i.e., cathode electrode upon the contralesional motor hemisphere to reduce inhibition of the ipsilesional hemisphere, and anode electrode upon the ipsilesional hemisphere to increase its excitability), while Tanaka et al. (2011) applied an unilateral motor tDCS configuration (i.e., anode electrode upon the ipsilesional motor hemisphere and cathode electrode upon the contralateral supraorbital area). Furthermore, some studies (Jeffery et al., 2007; Tanaka et al., 2011; Suzuki et al., 2012) used the TMS-motor evoked potential technique to locate the motor “hotspot” in leg muscle (i.e., vastus lateralis) before positioning tDCS electrodes over the subject’s scalp; unfortunately, we were not able to do so. Although C3/C4 electrode montage is acknowledged as adequate for eliciting modulation in lower limbs (Legatt et al., 2016), the low focality of tDCS stimulus induced by bihemispheric electrode montage to leg muscles may have attenuated the effects of tDCS on PT in both stroke and healthy groups. Secondly, Tanaka et al. (2011) assessed the force production using maximal isometric knee extension performed for 3 s, while in the present study three dynamic repetitions involving both extension and flexion of the quadriceps were applied. It is well accepted that antagonist restraint (in our case, the action of hamstrings) increases with movement velocity in maximal voluntary concentric efforts, due to exaggerated flexor stretch reflexes in the paretic limb (Knutsson et al., 1992). Thus, an exercise task involving maximal voluntary concentric movements could result in unfavorable increasing of antagonist contraction, which in our study might have attenuated the effects of tDCS upon the strength of knee extensors (Watkins et al., 1984).

A reduced force steadiness during submaximal motor tasks has been associated with motor impairment in post-stroke patients, due to lesions in connected areas within the ipsilesional hemisphere and lesions in interconnections with the contralesional hemisphere (Lodha et al., 2010; Westlake and Nagarajan, 2011). Even though non-paretic limbs might also exhibit decreased muscle power (Prado-Medeiros et al., 2012), a greater number of motor units seem to be recruited in paretic muscles to generate similar levels of force as in the non-paretic limb. Moreover, an increase of strength in the paretic limb seems to be correlated with a decrease in force variability (Chang et al., 2013). Our findings revealed that the post-stroke group improved their force steadiness during submaximal dynamic exercise in paretic and non-paretic limbs, after a single session of bihemispheric motor cortex tDCS. These data reinforce the findings of a previous study reporting greater improvements in ankle control, in response to anodal tDCS applied over the lesioned motor cortex during motor tasks (Madhavan et al., 2011). These beneficial effects would be possible due to a reduction in the intact-to-affected transcallosal inhibition, as well as to an increase in motor plasticity obtained by stimulating the affected motor hemisphere, favoring a better interhemispheric balance and more harmonic recruitment of motor units (Bolognini et al., 2011; Krishnan et al., 2014). Even though additional research is warranted to confirm these findings, this is an interesting and a useful possibility in terms of motor rehabilitation.

Recent studies failed to detect improvements in motor performance following tDCS (Kan et al., 2013; Montenegro et al., 2015), which has been attributed to a possible “ceiling effect” in the preserved motor cortex. Our results support this premise—considering that anode electrode was applied over the contralateral motor cortex, it was fairly expected that no difference in PT or force steadiness occurred in healthy controls. Furthermore, these results are consistent with previous studies reporting lower motor thresholds, higher motor-evoked potentials (De Gennaro et al., 2004), and shorter silent periods (Priori et al., 1999) in the motor cortex controlling dominant *vs.* non-dominant limbs. It could be therefore speculated that bihemispheric tDCS with anode electrode positioned over the non-dominant motor cortex would be an efficient strategy to increase strength, as suggested by a previous trial comparing hand function after stimulating non-dominant and dominant motor cortices of healthy subjects (Boggio et al., 2006).

Interestingly, our results showed non-specific effects due to tDCS on left and right legs in both groups. The tDCS electrode montage presently adopted (bihemispheric or dual tDCS) might help to explain this finding. When bilateral tDCS is applied, the cathode electrode is expected to reduce the motor control performance of the contralateral limb, whereas an improvement should be observed in the limb stimulated by the anode electrode. However, the tDCS effects upon a highly complex dynamic system like motor circuits do not always take place within a linear and predicted pattern of causality—adjustments and therefore unexpected outcomes might occur. In this sense, prior studies have observed decreased interhemispheric functional connectivity due to online bilateral primary motor tDCS, but also increased intracortical functional connectivity in areas placed below the anode electrode, concomitant to no effect in areas below the cathode electrode (Sehm et al., 2013). Hence it is feasible to think that the presently applied electrode montage was capable to attenuate the inhibitory influence that the dominant cortex exerted upon the lesioned area (transcallosal inhibition), which might have contributed to improve the intracortical functional connectivity and therefore the motor control in both limbs.

Based on this rationale, it is fair to speculate that stroke patients exhibiting interhemispheric imbalance might benefit from stimulation using bilateral tDCS configuration. Concisely, the favorable effects of tDCS upon motor control would be related to an attenuation of the unbalance between cortical hemispheres (Nowak et al., 2009), by reducing the inhibitory projections from the contralesional hemisphere (usually hyper excited in those patients), while increasing neuronal excitability of the compromised hemisphere (Sehm et al., 2013). This premise concurs, for instance, with the data from the study by Tahtis et al. (2014), demonstrating that gait performance improved after bilateral tDCS applied over cortical areas responsible for the control of lower limbs. Evidently, further research is warranted to confirm these possibilities.

Limitations of this study must be acknowledged. Firstly, specific cortical areas activated by tDCS were not controlled. Even though 10 min of 2 mA tDCS has been shown to increase leg corticospinal tract excitability for at least 60 min (Jeffery et al., 2007), the use of imaging techniques such as fMRI would be useful to ensure that targeted areas had been effectively stimulated. Secondly, the experimental groups were not age-matched (post-stroke patients aged 52 ± 14 year *vs.* healthy controls 26 ± 7 years), which has been suggested to influence the response to electric fields induced by motor cortex tDCS (Laakso et al., 2015). However, since the aging process seems to negatively affect the recruitment activation of brain areas during motor tasks (Manan et al., 2015), the choice of healthy young individuals as controls is justified to ensure that tDCS effects upon motor performance were tested across groups really exhibiting opposite status in regards to motor cortex integrity (e.g., healthy *vs.* lesioned).

In conclusion, a single session of tDCS applied over the motor cortex was able to improve force steadiness, but not maximal dynamic strength during a gross motor task (knee extension and flexion) in post-stroke hemiparetic patients. These findings suggest that tDCS might be considered as a complementary strategy to traditional rehabilitation training, in order to improve motor control and mobility of hemiparetic patients affected by stroke.

## Author Contributions

Conceived and designed the experiments: RAM, AHO, PF. Performed the experiments: RAM, RM, WB. Analyzed the data: RAM, AM, PF. Wrote the article: RAM, AM, AHO, PF, RM. Language review: AM.

## Funding

This study was supported by grants from the Brazilian Council for the Technological and Scientific Development (CNPq) and Carlos Chagas Filho Foundation for Research Support in the State of Rio de Janeiro (FAPERJ).

## Conflict of Interest Statement

The authors declare that the research was conducted in the absence of any commercial or financial relationships that could be construed as a potential conflict of interest.
